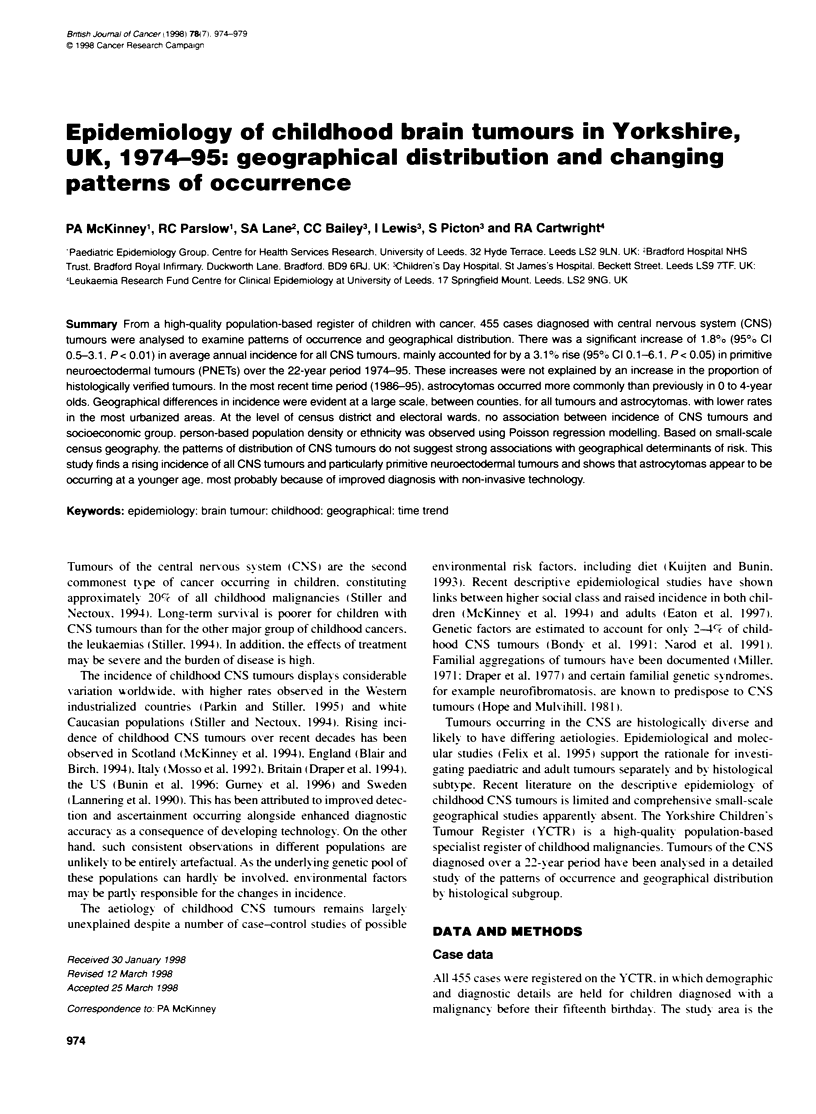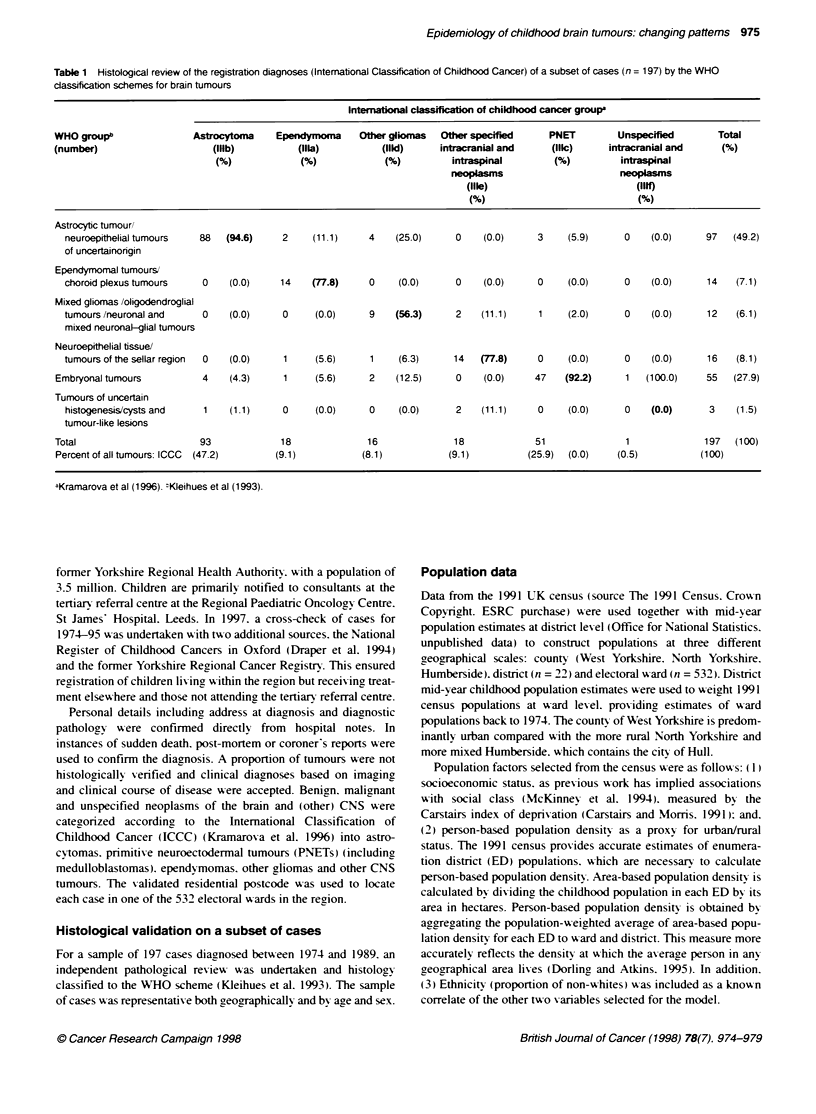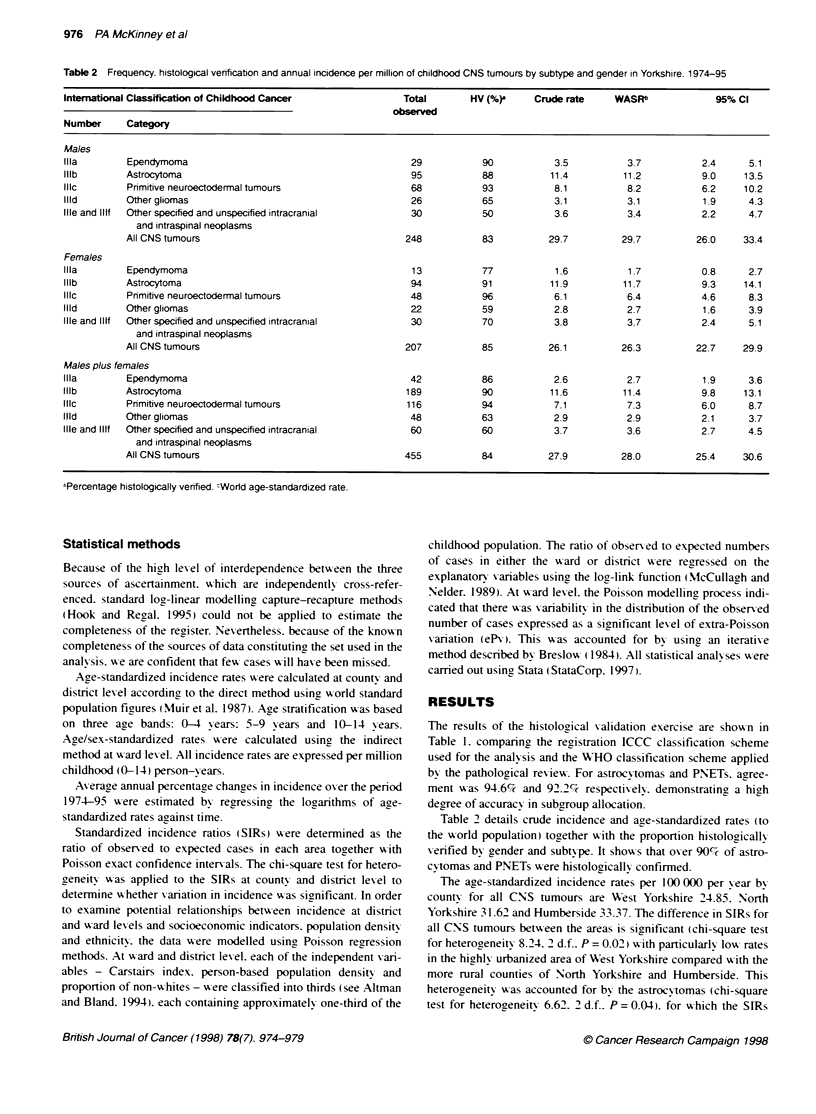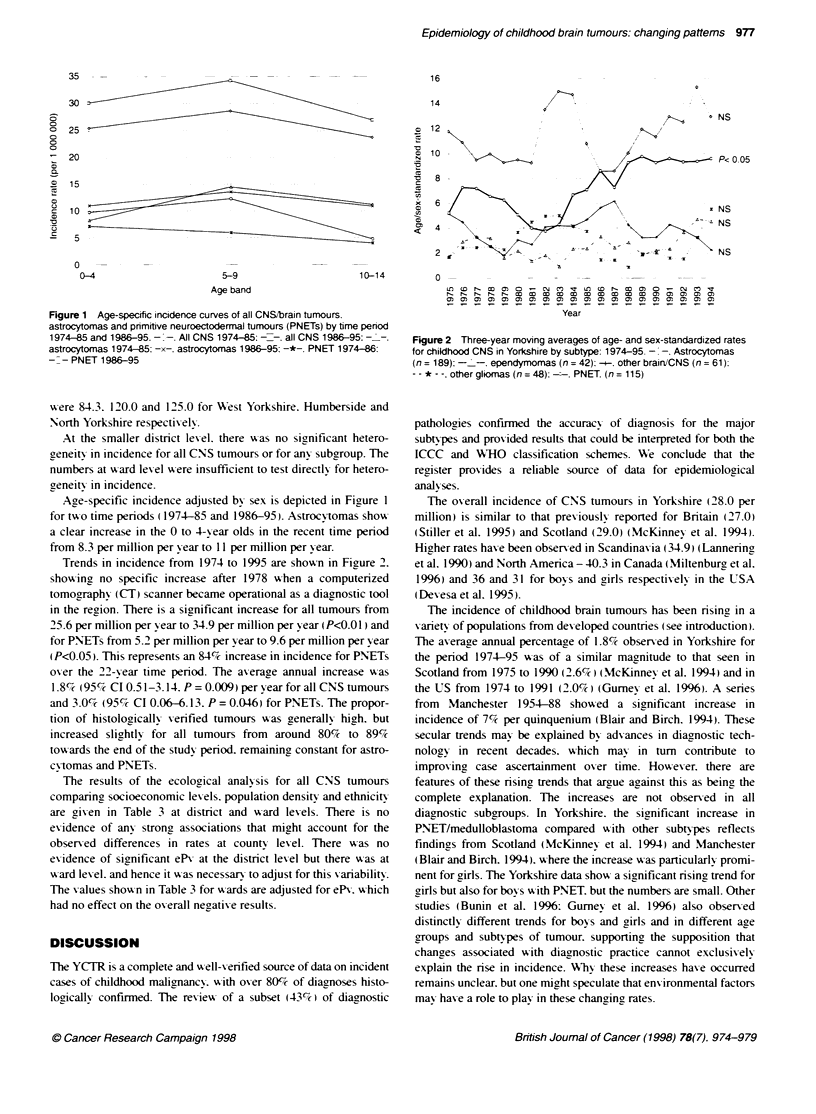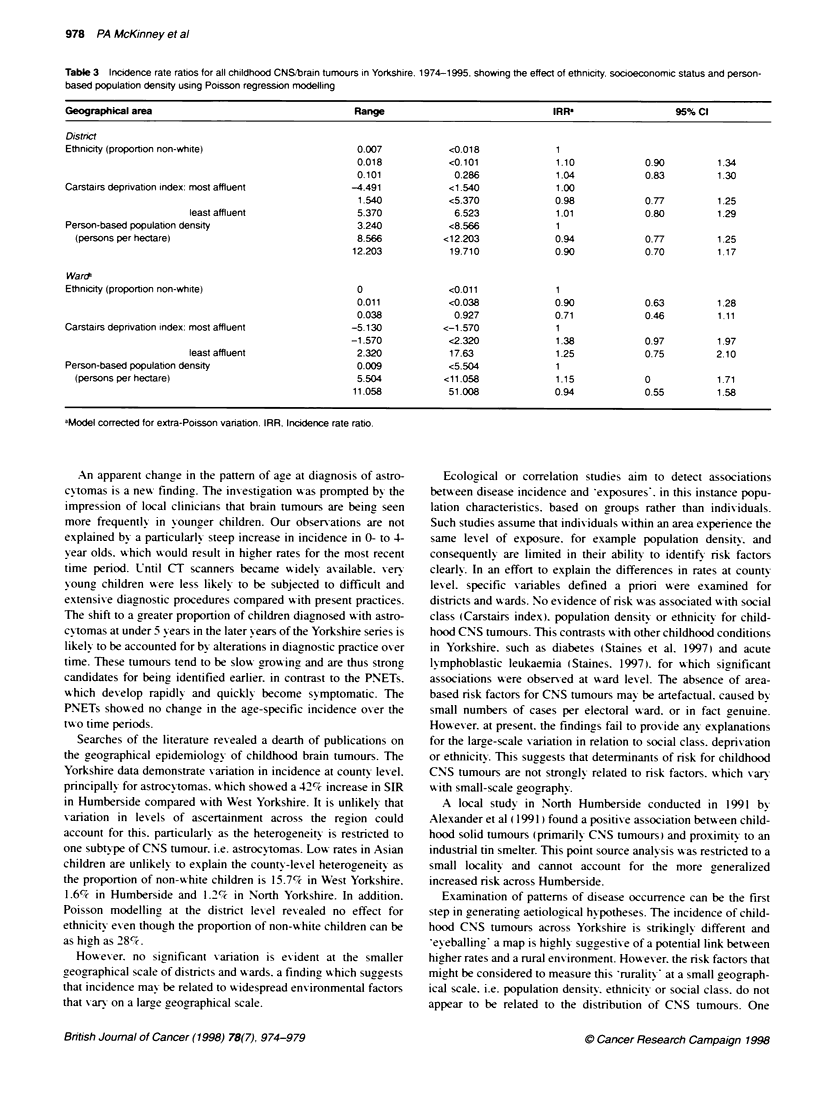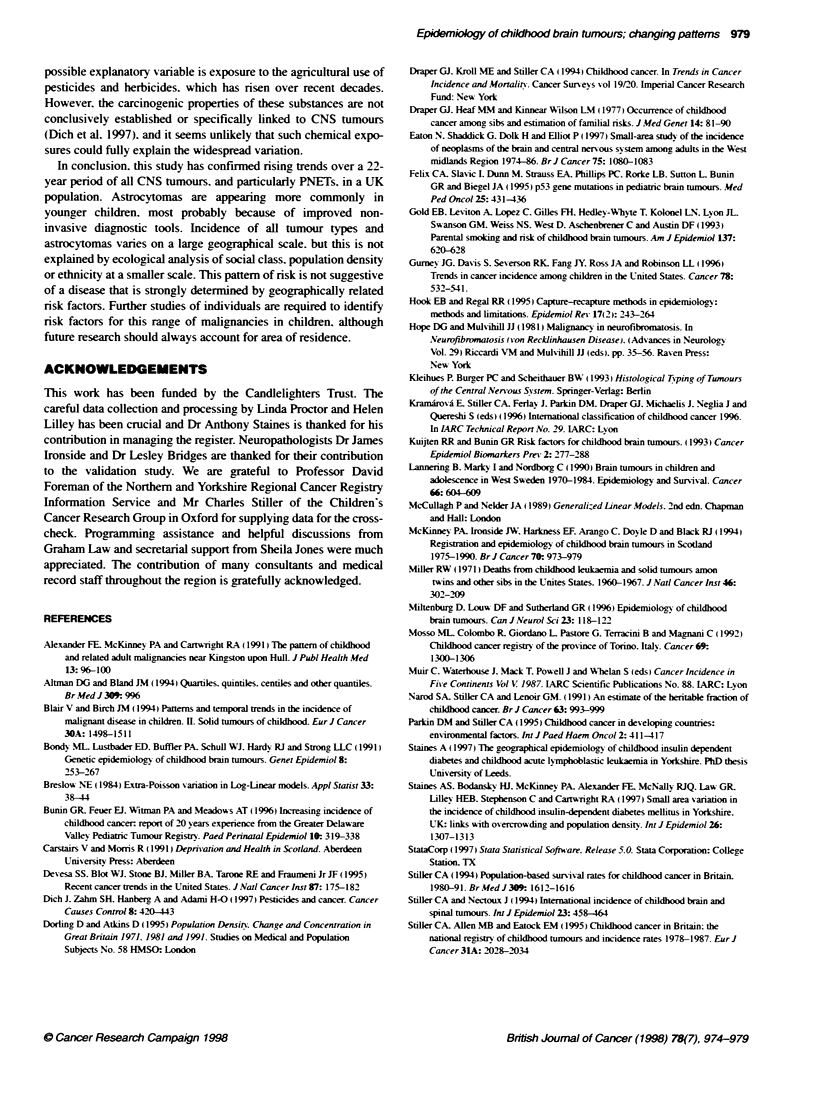# Epidemiology of childhood brain tumours in Yorkshire, UK, 1974-95: geographical distribution and changing patterns of occurrence.

**DOI:** 10.1038/bjc.1998.612

**Published:** 1998-10

**Authors:** P. A. McKinney, R. C. Parslow, S. A. Lane, C. C. Bailey, I. Lewis, S. Picton, R. A. Cartwright

**Affiliations:** Paediatric Epidemiology Group, Centre for Health Services Research, University of Leeds, UK.

## Abstract

From a high-quality population-based register of children with cancer, 455 cases diagnosed with central nervous system (CNS) tumours were analysed to examine patterns of occurrence and geographical distribution. There was a significant increase of 1.8% (95% CI 0.5-3.1, P < 0.01) in average annual incidence for all CNS tumours, mainly accounted for by a 3.1% rise (95% CI 0.1-6.1, P < 0.05) in primitive neuroectodermal tumours (PNETs) over the 22-year period 1974-95. These increases were not explained by an increase in the proportion of histologically verified tumours. In the most recent time period (1986-95), astrocytomas occurred more commonly than previously in 0 to 4-year olds. Geographical differences in incidence were evident at a large scale, between counties, for all tumours and astrocytomas, with lower rates in the most urbanized areas. At the level of census district and electoral wards, no association between incidence of CNS tumours and socioeconomic group, person-based population density or ethnicity was observed using Poisson regression modelling. Based on small-scale census geography, the patterns of distribution of CNS tumours do not suggest strong associations with geographical determinants of risk. This study finds a rising incidence of all CNS tumours and particularly primitive neuroectodermal tumours and shows that astrocytomas appear to be occurring at a younger age, most probably because of improved diagnosis with non-invasive technology.


British Journal of Cancer (1998) 78(7), 974–979
© 1998 Cancer Research Campaign

### Epidemiology of childhood brain tumours in Yorkshire, UK, 1974–95: geographical distribution and changing patterns of occurrence

**PA McKinney[1], RC Parslow[1], SA Lane[2], CC Bailey[3], I Lewis[3], S Picton[3] and RA Cartwright[4]**

[1]Paediatric Epidemiology Group, Centre for Health Services Research, University of Leeds, 32 Hyde Terrace, Leeds LS2 9LN, UK; [2]Bradford Hospital NHS Trust, Bradford Royal Infirmary, Duckworth Lane, Bradford, BD9 6RJ, UK; [3]Children's Day Hospital, St James's Hospital, Beckett Street, Leeds LS9 7TF, UK; [4]Leukaemia Research Fund Centre for Clinical Epidemiology at University of Leeds, 17 Springfield Mount, Leeds, LS2 9NG, UK

**Summary** From a high-quality population-based register of children with cancer, 455 cases diagnosed with central nervous system (CNS) tumours were analysed to examine patterns of occurrence and geographical distribution. There was a significant increase of 1.8% (95% CI 0.5–3.1, $P < 0.01$) in average annual incidence for all CNS tumours, mainly accounted for by a 3.1% rise (95% CI 0.1–6.1, $P < 0.05$) in primitive neuroectodermal tumours (PNETs) over the 22-year period 1974–95. These increases were not explained by an increase in the proportion of histologically verified tumours. In the most recent time period (1986–95), astrocytomas occurred more commonly than previously in 0 to 4-year olds. Geographical differences in incidence were evident at a large scale, between counties, for all tumours and astrocytomas, with lower rates in the most urbanized areas. At the level of census district and electoral wards, no association between incidence of CNS tumours and socioeconomic group, person-based population density or ethnicity was observed using Poisson regression modelling. Based on small-scale census geography, the patterns of distribution of CNS tumours do not suggest strong associations with geographical determinants of risk. This study finds a rising incidence of all CNS tumours and particularly primitive neuroectodermal tumours and shows that astrocytomas appear to be occurring at a younger age, most probably because of improved diagnosis with non-invasive technology.

**Keywords:** epidemiology; brain tumour; childhood; geographical; time trend

Tumours of the central nervous system (CNS) are the second commonest type of cancer occurring in children, constituting approximately 20% of all childhood malignancies (Stiller and Nectoux, 1994). Long-term survival is poorer for children with CNS tumours than for the other major group of childhood cancers, the leukaemias (Stiller, 1994). In addition, the effects of treatment may be severe and the burden of disease is high.

The incidence of childhood CNS tumours displays considerable variation worldwide, with higher rates observed in the Western industrialized countries (Parkin and Stiller, 1995) and white Caucasian populations (Stiller and Nectoux, 1994). Rising incidence of childhood CNS tumours over recent decades has been observed in Scotland (McKinney et al, 1994), England (Blair and Birch, 1994), Italy (Mosso et al, 1992), Britain (Draper et al, 1994), the US (Bunin et al, 1996; Gurney et al, 1996) and Sweden (Lannering et al, 1990). This has been attributed to improved detection and ascertainment occurring alongside enhanced diagnostic accuracy as a consequence of developing technology. On the other hand, such consistent observations in different populations are unlikely to be entirely artefactual. As the underlying genetic pool of these populations can hardly be involved, environmental factors may be partly responsible for the changes in incidence.

The aetiology of childhood CNS tumours remains largely unexplained despite a number of case–control studies of possible environmental risk factors, including diet (Kuijten and Bunin, 1993). Recent descriptive epidemiological studies have shown links between higher social class and raised incidence in both children (McKinney et al, 1994) and adults (Eaton et al, 1997). Genetic factors are estimated to account for only 2–4% of childhood CNS tumours (Bondy et al, 1991; Narod et al, 1991). Familial aggregations of tumours have been documented (Miller, 1971; Draper et al, 1977) and certain familial genetic syndromes, for example neurofibromatosis, are known to predispose to CNS tumours (Hope and Mulvihill, 1981).

Tumours occurring in the CNS are histologically diverse and likely to have differing aetiologies. Epidemiological and molecular studies (Felix et al, 1995) support the rationale for investigating paediatric and adult tumours separately and by histological subtype. Recent literature on the descriptive epidemiology of childhood CNS tumours is limited and comprehensive small-scale geographical studies apparently absent. The Yorkshire Children's Tumour Register (YCTR) is a high-quality population-based specialist register of childhood malignancies. Tumours of the CNS diagnosed over a 22-year period have been analysed in a detailed study of the patterns of occurrence and geographical distribution by histological subgroup.

### DATA AND METHODS

### Case data

All 455 cases were registered on the YCTR, in which demographic and diagnostic details are held for children diagnosed with a malignancy before their fifteenth birthday. The study area is the

*Received 30 January 1998*
*Revised 12 March 1998*
*Accepted 25 March 1998*

*Correspondence to:* PA McKinney

**Table 1** Histological review of the registration diagnoses (International Classification of Childhood Cancer) of a subset of cases (*n* = 197) by the WHO classification schemes for brain tumours

| WHO group[b] (number) | International classification of childhood cancer group[a] Astrocytoma (IIIb) (%) | | Ependymoma (IIIa) (%) | | Other gliomas (IIId) (%) | | Other specified intracranial and intraspinal neoplasms (IIIe) (%) | | PNET (IIIc) (%) | | Unspecified intracranial and intraspinal neoplasms (IIIf) (%) | | Total (%) | |
|---|---|---|---|---|---|---|---|---|---|---|---|---|---|---|
| Astrocytic tumour/ neuroepithelial tumours of uncertainorigin | 88 | **(94.6)** | 2 | (11.1) | 4 | (25.0) | 0 | (0.0) | 3 | (5.9) | 0 | (0.0) | 97 | (49.2) |
| Ependymomal tumours/ choroid plexus tumours | 0 | (0.0) | 14 | **(77.8)** | 0 | (0.0) | 0 | (0.0) | 0 | (0.0) | 0 | (0.0) | 14 | (7.1) |
| Mixed gliomas /oligodendroglial tumours /neuronal and mixed neuronal–glial tumours | 0 | (0.0) | 0 | (0.0) | 9 | **(56.3)** | 2 | (11.1) | 1 | (2.0) | 0 | (0.0) | 12 | (6.1) |
| Neuroepithelial tissue/ tumours of the sellar region | 0 | (0.0) | 1 | (5.6) | 1 | (6.3) | 14 | **(77.8)** | 0 | (0.0) | 0 | (0.0) | 16 | (8.1) |
| Embryonal tumours | 4 | (4.3) | 1 | (5.6) | 2 | (12.5) | 0 | (0.0) | 47 | **(92.2)** | 1 | (100.0) | 55 | (27.9) |
| Tumours of uncertain histogenesis/cysts and tumour-like lesions | 1 | (1.1) | 0 | (0.0) | 0 | (0.0) | 2 | (11.1) | 0 | (0.0) | 0 | **(0.0)** | 3 | (1.5) |
| Total | 93 | | 18 | | 16 | | 18 | | 51 | | 1 | | 197 | (100) |
| Percent of all tumours: ICCC | (47.2) | | (9.1) | | (8.1) | | (9.1) | | (25.9) | (0.0) | (0.5) | | (100) | |

[a]Kramarova et al (1996). [b]Kleihues et al (1993).

former Yorkshire Regional Health Authority, with a population of 3.5 million. Children are primarily notified to consultants at the tertiary referral centre at the Regional Paediatric Oncology Centre, St James' Hospital, Leeds. In 1997, a cross-check of cases for 1974–95 was undertaken with two additional sources, the National Register of Childhood Cancers in Oxford (Draper et al, 1994) and the former Yorkshire Regional Cancer Registry. This ensured registration of children living within the region but receiving treatment elsewhere and those not attending the tertiary referral centre.

Personal details including address at diagnosis and diagnostic pathology were confirmed directly from hospital notes. In instances of sudden death, post-mortem or coroner's reports were used to confirm the diagnosis. A proportion of tumours were not histologically verified and clinical diagnoses based on imaging and clinical course of disease were accepted. Benign, malignant and unspecified neoplasms of the brain and (other) CNS were categorized according to the International Classification of Childhood Cancer (ICCC) (Kramarova et al, 1996) into astrocytomas, primitive neuroectodermal tumours (PNETs) (including medulloblastomas), ependymomas, other gliomas and other CNS tumours. The validated residential postcode was used to locate each case in one of the 532 electoral wards in the region.

### Histological validation on a subset of cases

For a sample of 197 cases diagnosed between 1974 and 1989, an independent pathological review was undertaken and histology classified to the WHO scheme (Kleihues et al, 1993). The sample of cases was representative both geographically and by age and sex.

### Population data

Data from the 1991 UK census (source The 1991 Census, Crown Copyright, ESRC purchase) were used together with mid-year population estimates at district level (Office for National Statistics, unpublished data) to construct populations at three different geographical scales: county (West Yorkshire, North Yorkshire, Humberside), district (*n* = 22) and electoral ward (*n* = 532). District mid-year childhood population estimates were used to weight 1991 census populations at ward level, providing estimates of ward populations back to 1974. The county of West Yorkshire is predominantly urban compared with the more rural North Yorkshire and more mixed Humberside, which contains the city of Hull.

Population factors selected from the census were as follows: (1) socioeconomic status, as previous work has implied associations with social class (McKinney et al, 1994), measured by the Carstairs index of deprivation (Carstairs and Morris, 1991); and, (2) person-based population density as a proxy for urban/rural status. The 1991 census provides accurate estimates of enumeration district (ED) populations, which are necessary to calculate person-based population density. Area-based population density is calculated by dividing the childhood population in each ED by its area in hectares. Person-based population density is obtained by aggregating the population-weighted average of area-based population density for each ED to ward and district. This measure more accurately reflects the density at which the average person in any geographical area lives (Dorling and Atkins, 1995). In addition, (3) Ethnicity (proportion of non-whites) was included as a known correlate of the other two variables selected for the model.

© Cancer Research Campaign 1998

**Table 2** Frequency, histological verification and annual incidence per million of childhood CNS tumours by subtype and gender in Yorkshire, 1974–95

| International Classification of Childhood Cancer | | Total observed | HV (%)[a] | Crude rate | WASR[b] | 95% CI | |
|---|---|---|---|---|---|---|---|
| Number | Category | | | | | | |
| *Males* | | | | | | | |
| IIIa | Ependymoma | 29 | 90 | 3.5 | 3.7 | 2.4 | 5.1 |
| IIIb | Astrocytoma | 95 | 88 | 11.4 | 11.2 | 9.0 | 13.5 |
| IIIc | Primitive neuroectodermal tumours | 68 | 93 | 8.1 | 8.2 | 6.2 | 10.2 |
| IIId | Other gliomas | 26 | 65 | 3.1 | 3.1 | 1.9 | 4.3 |
| IIIe and IIIf | Other specified and unspecified intracranial and intraspinal neoplasms | 30 | 50 | 3.6 | 3.4 | 2.2 | 4.7 |
| | All CNS tumours | 248 | 83 | 29.7 | 29.7 | 26.0 | 33.4 |
| *Females* | | | | | | | |
| IIIa | Ependymoma | 13 | 77 | 1.6 | 1.7 | 0.8 | 2.7 |
| IIIb | Astrocytoma | 94 | 91 | 11.9 | 11.7 | 9.3 | 14.1 |
| IIIc | Primitive neuroectodermal tumours | 48 | 96 | 6.1 | 6.4 | 4.6 | 8.3 |
| IIId | Other gliomas | 22 | 59 | 2.8 | 2.7 | 1.6 | 3.9 |
| IIIe and IIIf | Other specified and unspecified intracranial and intraspinal neoplasms | 30 | 70 | 3.8 | 3.7 | 2.4 | 5.1 |
| | All CNS tumours | 207 | 85 | 26.1 | 26.3 | 22.7 | 29.9 |
| *Males plus females* | | | | | | | |
| IIIa | Ependymoma | 42 | 86 | 2.6 | 2.7 | 1.9 | 3.6 |
| IIIb | Astrocytoma | 189 | 90 | 11.6 | 11.4 | 9.8 | 13.1 |
| IIIc | Primitive neuroectodermal tumours | 116 | 94 | 7.1 | 7.3 | 6.0 | 8.7 |
| IIId | Other gliomas | 48 | 63 | 2.9 | 2.9 | 2.1 | 3.7 |
| IIIe and IIIf | Other specified and unspecified intracranial and intraspinal neoplasms | 60 | 60 | 3.7 | 3.6 | 2.7 | 4.5 |
| | All CNS tumours | 455 | 84 | 27.9 | 28.0 | 25.4 | 30.6 |

[a]Percentage histologically verified. [b]World age-standardized rate.

### Statistical methods

Because of the high level of interdependence between the three sources of ascertainment, which are independently cross-referenced, standard log-linear modelling capture–recapture methods (Hook and Regal, 1995) could not be applied to estimate the completeness of the register. Nevertheless, because of the known completeness of the sources of data constituting the set used in the analysis, we are confident that few cases will have been missed.

Age-standardized incidence rates were calculated at county and district level according to the direct method using world standard population figures (Muir et al, 1987). Age stratification was based on three age bands: 0–4 years; 5–9 years and 10–14 years. Age/sex-standardized rates were calculated using the indirect method at ward level. All incidence rates are expressed per million childhood (0–14) person-years.

Average annual percentage changes in incidence over the period 1974–95 were estimated by regressing the logarithms of age-standardized rates against time.

Standardized incidence ratios (SIRs) were determined as the ratio of observed to expected cases in each area together with Poisson exact confidence intervals. The chi-square test for heterogeneity was applied to the SIRs at county and district level to determine whether variation in incidence was significant. In order to examine potential relationships between incidence at district and ward levels and socioeconomic indicators, population density and ethnicity, the data were modelled using Poisson regression methods. At ward and district level, each of the independent variables – Carstairs index, person-based population density and proportion of non-whites – were classified into thirds (see Altman and Bland, 1994), each containing approximately one-third of the childhood population. The ratio of observed to expected numbers of cases in either the ward or district were regressed on the explanatory variables using the log-link function (McCullagh and Nelder, 1989). At ward level, the Poisson modelling process indicated that there was variability in the distribution of the observed number of cases expressed as a significant level of extra-Poisson variation (ePv). This was accounted for by using an iterative method described by Breslow (1984). All statistical analyses were carried out using Stata (StataCorp, 1997).

### RESULTS

The results of the histological validation exercise are shown in Table 1, comparing the registration ICCC classification scheme used for the analysis and the WHO classification scheme applied by the pathological review. For astrocytomas and PNETs, agreement was 94.6% and 92.2% respectively, demonstrating a high degree of accuracy in subgroup allocation.

Table 2 details crude incidence and age-standardized rates (to the world population) together with the proportion histologically verified by gender and subtype. It shows that over 90% of astrocytomas and PNETs were histologically confirmed.

The age-standardized incidence rates per 100 000 per year by county for all CNS tumours are West Yorkshire 24.85, North Yorkshire 31.62 and Humberside 33.37. The difference in SIRs for all CNS tumours between the areas is significant (chi-square test for heterogeneity 8.24, 2 d.f., $P = 0.02$) with particularly low rates in the highly urbanized area of West Yorkshire compared with the more rural counties of North Yorkshire and Humberside. This heterogeneity was accounted for by the astrocytomas (chi-square test for heterogeneity 6.62, 2 d.f., $P = 0.04$), for which the SIRs

© Cancer Research Campaign 1998

**Figure 1** Age-specific incidence curves of all CNS/brain tumours. astrocytomas and primitive neuroectodermal tumours (PNETs) by time period 1974–85 and 1986–95. –◦–. All CNS 1974–85: –□–. all CNS 1986–95: –△–. astrocytomas 1974–85: –x–. astrocytomas 1986–95: –★–. PNET 1974–86: –□– PNET 1986–95

**Figure 2** Three-year moving averages of age- and sex-standardized rates for childhood CNS in Yorkshire by subtype: 1974–95. –◦–. Astrocytomas ($n$ = 189): –△–. ependymomas ($n$ = 42): –+–. other brain/CNS ($n$ = 61): - - ★ - -. other gliomas ($n$ = 48): –◦–. PNET. ($n$ = 115)

were 84.3. 120.0 and 125.0 for West Yorkshire. Humberside and North Yorkshire respectively.

At the smaller district level. there was no significant heterogeneity in incidence for all CNS tumours or for any subgroup. The numbers at ward level were insufficient to test directly for heterogeneity in incidence.

Age-specific incidence adjusted by sex is depicted in Figure 1 for two time periods (1974–85 and 1986–95). Astrocytomas show a clear increase in the 0 to 4-year olds in the recent time period from 8.3 per million per year to 11 per million per year.

Trends in incidence from 1974 to 1995 are shown in Figure 2. showing no specific increase after 1978 when a computerized tomography (CT) scanner became operational as a diagnostic tool in the region. There is a significant increase for all tumours from 25.6 per million per year to 34.9 per million per year ($P$<0.01) and for PNETs from 5.2 per million per year to 9.6 per million per year ($P$<0.05). This represents an 84% increase in incidence for PNETs over the 22-year time period. The average annual increase was 1.8% (95% CI 0.51–3.14. $P$ = 0.009) per year for all CNS tumours and 3.0% (95% CI 0.06–6.13. $P$ = 0.046) for PNETs. The proportion of histologically verified tumours was generally high. but increased slightly for all tumours from around 80% to 89% towards the end of the study period. remaining constant for astrocytomas and PNETs.

The results of the ecological analysis for all CNS tumours comparing socioeconomic levels. population density and ethnicity are given in Table 3 at district and ward levels. There is no evidence of any strong associations that might account for the observed differences in rates at county level. There was no evidence of significant ePv at the district level but there was at ward level. and hence it was necessary to adjust for this variability. The values shown in Table 3 for wards are adjusted for ePv. which had no effect on the overall negative results.

### DISCUSSION

The YCTR is a complete and well-verified source of data on incident cases of childhood malignancy. with over 80% of diagnoses histologically confirmed. The review of a subset (43%) of diagnostic pathologies confirmed the accuracy of diagnosis for the major subtypes and provided results that could be interpreted for both the ICCC and WHO classification schemes. We conclude that the register provides a reliable source of data for epidemiological analyses.

The overall incidence of CNS tumours in Yorkshire (28.0 per million) is similar to that previously reported for Britain (27.0) (Stiller et al. 1995) and Scotland (29.0) (McKinney et al. 1994). Higher rates have been observed in Scandinavia (34.9) (Lannering et al. 1990) and North America – 40.3 in Canada (Miltenburg et al. 1996) and 36 and 31 for boys and girls respectively in the USA (Devesa et al. 1995).

The incidence of childhood brain tumours has been rising in a variety of populations from developed countries (see introduction). The average annual percentage of 1.8% observed in Yorkshire for the period 1974–95 was of a similar magnitude to that seen in Scotland from 1975 to 1990 (2.6%) (McKinney et al. 1994) and in the US from 1974 to 1991 (2.0%) (Gurney et al. 1996). A series from Manchester 1954–88 showed a significant increase in incidence of 7% per quinquenium (Blair and Birch. 1994). These secular trends may be explained by advances in diagnostic technology in recent decades. which may in turn contribute to improving case ascertainment over time. However. there are features of these rising trends that argue against this as being the complete explanation. The increases are not observed in all diagnostic subgroups. In Yorkshire. the significant increase in PNET/medulloblastoma compared with other subtypes reflects findings from Scotland (McKinney et al. 1994) and Manchester (Blair and Birch. 1994). where the increase was particularly prominent for girls. The Yorkshire data show a significant rising trend for girls but also for boys with PNET. but the numbers are small. Other studies (Bunin et al. 1996: Gurney et al. 1996) also observed distinctly different trends for boys and girls and in different age groups and subtypes of tumour. supporting the supposition that changes associated with diagnostic practice cannot exclusively explain the rise in incidence. Why these increases have occurred remains unclear. but one might speculate that environmental factors may have a role to play in these changing rates.

© Cancer Research Campaign 1998

**Table 3** Incidence rate ratios for all childhood CNS/brain tumours in Yorkshire. 1974–1995. showing the effect of ethnicity. socioeconomic status and person-based population density using Poisson regression modelling

| Geographical area | Range | | IRR[a] | 95% CI | |
|---|---|---|---|---|---|
| *District* | | | | | |
| Ethnicity (proportion non-white) | 0.007 | <0.018 | 1 | | |
| | 0.018 | <0.101 | 1.10 | 0.90 | 1.34 |
| | 0.101 | 0.286 | 1.04 | 0.83 | 1.30 |
| Carstairs deprivation index: most affluent | –4.491 | <1.540 | 1.00 | | |
| | 1.540 | <5.370 | 0.98 | 0.77 | 1.25 |
| least affluent | 5.370 | 6.523 | 1.01 | 0.80 | 1.29 |
| Person-based population density | 3.240 | <8.566 | 1 | | |
| (persons per hectare) | 8.566 | <12.203 | 0.94 | 0.77 | 1.25 |
| | 12.203 | 19.710 | 0.90 | 0.70 | 1.17 |
| *Ward*[b] | | | | | |
| Ethnicity (proportion non-white) | 0 | <0.011 | 1 | | |
| | 0.011 | <0.038 | 0.90 | 0.63 | 1.28 |
| | 0.038 | 0.927 | 0.71 | 0.46 | 1.11 |
| Carstairs deprivation index: most affluent | –5.130 | <–1.570 | 1 | | |
| | –1.570 | <2.320 | 1.38 | 0.97 | 1.97 |
| least affluent | 2.320 | 17.63 | 1.25 | 0.75 | 2.10 |
| Person-based population density | 0.009 | <5.504 | 1 | | |
| (persons per hectare) | 5.504 | <11.058 | 1.15 | 0 | 1.71 |
| | 11.058 | 51.008 | 0.94 | 0.55 | 1.58 |

[a]Model corrected for extra-Poisson variation. IRR. Incidence rate ratio.

An apparent change in the pattern of age at diagnosis of astrocytomas is a new finding. The investigation was prompted by the impression of local clinicians that brain tumours are being seen more frequently in younger children. Our observations are not explained by a particularly steep increase in incidence in 0- to 4-year olds. which would result in higher rates for the most recent time period. Until CT scanners became widely available. very young children were less likely to be subjected to difficult and extensive diagnostic procedures compared with present practices. The shift to a greater proportion of children diagnosed with astrocytomas at under 5 years in the later years of the Yorkshire series is likely to be accounted for by alterations in diagnostic practice over time. These tumours tend to be slow growing and are thus strong candidates for being identified earlier. in contrast to the PNETs. which develop rapidly and quickly become symptomatic. The PNETs showed no change in the age-specific incidence over the two time periods.

Searches of the literature revealed a dearth of publications on the geographical epidemiology of childhood brain tumours. The Yorkshire data demonstrate variation in incidence at county level. principally for astrocytomas. which showed a 42% increase in SIR in Humberside compared with West Yorkshire. It is unlikely that variation in levels of ascertainment across the region could account for this. particularly as the heterogeneity is restricted to one subtype of CNS tumour. i.e. astrocytomas. Low rates in Asian children are unlikely to explain the county-level heterogeneity as the proportion of non-white children is 15.7% in West Yorkshire. 1.6% in Humberside and 1.2% in North Yorkshire. In addition. Poisson modelling at the district level revealed no effect for ethnicity even though the proportion of non-white children can be as high as 28%.

However. no significant variation is evident at the smaller geographical scale of districts and wards. a finding which suggests that incidence may be related to widespread environmental factors that vary on a large geographical scale.

Ecological or correlation studies aim to detect associations between disease incidence and 'exposures'. in this instance population characteristics. based on groups rather than individuals. Such studies assume that individuals within an area experience the same level of exposure. for example population density. and consequently are limited in their ability to identify risk factors clearly. In an effort to explain the differences in rates at county level. specific variables defined a priori were examined for districts and wards. No evidence of risk was associated with social class (Carstairs index). population density or ethnicity for childhood CNS tumours. This contrasts with other childhood conditions in Yorkshire. such as diabetes (Staines et al. 1997) and acute lymphoblastic leukaemia (Staines. 1997). for which significant associations were observed at ward level. The absence of area-based risk factors for CNS tumours may be artefactual. caused by small numbers of cases per electoral ward. or in fact genuine. However. at present. the findings fail to provide any explanations for the large-scale variation in relation to social class. deprivation or ethnicity. This suggests that determinants of risk for childhood CNS tumours are not strongly related to risk factors. which vary with small-scale geography.

A local study in North Humberside conducted in 1991 by Alexander et al (1991) found a positive association between childhood solid tumours (primarily CNS tumours) and proximity to an industrial tin smelter. This point source analysis was restricted to a small locality and cannot account for the more generalized increased risk across Humberside.

Examination of patterns of disease occurrence can be the first step in generating aetiological hypotheses. The incidence of childhood CNS tumours across Yorkshire is strikingly different and 'eyeballing' a map is highly suggestive of a potential link between higher rates and a rural environment. However. the risk factors that might be considered to measure this 'rurality' at a small geographical scale. i.e. population density. ethnicity or social class. do not appear to be related to the distribution of CNS tumours. One

*© Cancer Research Campaign 1998*

possible explanatory variable is exposure to the agricultural use of pesticides and herbicides, which has risen over recent decades. However, the carcinogenic properties of these substances are not conclusively established or specifically linked to CNS tumours (Dich et al. 1997), and it seems unlikely that such chemical exposures could fully explain the widespread variation.

In conclusion, this study has confirmed rising trends over a 22-year period of all CNS tumours, and particularly PNETs, in a UK population. Astrocytomas are appearing more commonly in younger children, most probably because of improved non-invasive diagnostic tools. Incidence of all tumour types and astrocytomas varies on a large geographical scale, but this is not explained by ecological analysis of social class, population density or ethnicity at a smaller scale. This pattern of risk is not suggestive of a disease that is strongly determined by geographically related risk factors. Further studies of individuals are required to identify risk factors for this range of malignancies in children, although future research should always account for area of residence.

### ACKNOWLEDGEMENTS

This work has been funded by the Candlelighters Trust. The careful data collection and processing by Linda Proctor and Helen Lilley has been crucial and Dr Anthony Staines is thanked for his contribution in managing the register. Neuropathologists Dr James Ironside and Dr Lesley Bridges are thanked for their contribution to the validation study. We are grateful to Professor David Foreman of the Northern and Yorkshire Regional Cancer Registry Information Service and Mr Charles Stiller of the Children's Cancer Research Group in Oxford for supplying data for the cross-check. Programming assistance and helpful discussions from Graham Law and secretarial support from Sheila Jones were much appreciated. The contribution of many consultants and medical record staff throughout the region is gratefully acknowledged.

### REFERENCES

Alexander FE, McKinney PA and Cartwright RA (1991) The pattern of childhood and related adult malignancies near Kingston upon Hull. *J Publ Health Med* **13:** 96–100

Altman DG and Bland JM (1994) Quartiles, quintiles, centiles and other quantiles. *Br Med J* **309:** 996

Blair V and Birch JM (1994) Patterns and temporal trends in the incidence of malignant disease in children. II. Solid tumours of childhood. *Eur J Cancer* **30A:** 1498–1511

Bondy ML, Lustbader ED, Buffler PA, Schull WJ, Hardy RJ and Strong LLC (1991) Genetic epidemiology of childhood brain tumours. *Genet Epidemiol* **8:** 253–267

Breslow NE (1984) Extra-Poisson variation in Log-Linear models. *Appl Statist* **33:** 38–44

Bunin GR, Feuer EJ, Witman PA and Meadows AT (1996) Increasing incidence of childhood cancer: report of 20 years experience from the Greater Delaware Valley Pediatric Tumour Registry. *Paed Perinatal Epidemiol* **10:** 319–338

Carstairs V and Morris R (1991) *Deprivation and Health in Scotland*. Aberdeen University Press: Aberdeen

Devesa SS, Blot WJ, Stone BJ, Miller BA, Tarone RE and Fraumeni Jr JF (1995) Recent cancer trends in the United States. *J Natl Cancer Inst* **87:** 175–182

Dich J, Zahm SH, Hanberg A and Adami H-O (1997) Pesticides and cancer. *Cancer Causes Control* **8:** 420–443

Dorling D and Atkins D (1995) *Population Density, Change and Concentration in Great Britain 1971, 1981 and 1991*. Studies on Medical and Population Subjects No. 58 HMSO: London

Draper GJ, Kroll ME and Stiller CA (1994) Childhood cancer. In *Trends in Cancer Incidence and Mortality*. Cancer Surveys vol 19/20. Imperial Cancer Research Fund: New York

Draper GJ, Heaf MM and Kinnear Wilson LM (1977) Occurrence of childhood cancer among sibs and estimation of familial risks. *J Med Genet* **14:** 81–90

Eaton N, Shaddick G, Dolk H and Elliot P (1997) Small-area study of the incidence of neoplasms of the brain and central nervous system among adults in the West midlands Region 1974–86. *Br J Cancer* **75:** 1080–1083

Felix CA, Slavic I, Dunn M, Strauss EA, Phillips PC, Rorke LB, Sutton L, Bunin GR and Biegel JA (1995) p53 gene mutations in pediatric brain tumours. *Med Ped Oncol* **25:** 431–436

Gold EB, Leviton A, Lopez C, Gilles FH, Hedley-Whyte T, Kolonel LN, Lyon JL, Swanson GM, Weiss NS, West D, Aschenbrener C and Austin DF (1993) Parental smoking and risk of childhood brain tumours. *Am J Epidemiol* **137:** 620–628

Gurney JG, Davis S, Severson RK, Fang JY, Ross JA and Robinson LL (1996) Trends in cancer incidence among children in the United States. *Cancer* **78:** 532–541.

Hook EB and Regal RR (1995) Capture–recapture methods in epidemiology: methods and limitations. *Epidemiol Rev* **17**(2): 243–264

Hope DG and Mulvihill JJ (1981) Malignancy in neurofibromatosis. In *Neurofibromatosis (von Recklinhausen Disease)*. (Advances in Neurology Vol. 29) Riccardi VM and Mulvihill JJ (eds), pp. 35–56. Raven Press: New York

Kleihues P, Burger PC and Scheithauer BW (1993) *Histological Typing of Tumours of the Central Nervous System*. Springer-Verlag: Berlin

Kramárová E, Stiller CA, Ferlay J, Parkin DM, Draper GJ, Michaelis J, Neglia J and Quereshi S (eds) (1996) International classification of childhood cancer 1996. In *IARC Technical Report No. 29*. IARC: Lyon

Kuijten RR and Bunin GR Risk factors for childhood brain tumours. (1993) *Cancer Epidemiol Biomarkers Prev* **2:** 277–288

Lannering B, Marky I and Nordborg C (1990) Brain tumours in children and adolescence in West Sweden 1970–1984. Epidemiology and Survival. *Cancer* **66:** 604–609

McCullagh P and Nelder JA (1989) *Generalized Linear Models*. 2nd edn. Chapman and Hall: London

McKinney PA, Ironside JW, Harkness EF, Arango C, Doyle D and Black RJ (1994) Registration and epidemiology of childhood brain tumours in Scotland 1975–1990. *Br J Cancer* **70:** 973–979

Miller RW (1971) Deaths from childhood leukaemia and solid tumours amon twins and other sibs in the Unites States, 1960–1967. *J Natl Cancer Inst* **46:** 302–209

Miltenburg D, Louw DF and Sutherland GR (1996) Epidemiology of childhood brain tumours. *Can J Neurol Sci* **23:** 118–122

Mosso ML, Colombo R, Giordano L, Pastore G, Terracini B and Magnani C (1992) Childhood cancer registry of the province of Torino, Italy. *Cancer* **69:** 1300–1306

Muir C, Waterhouse J, Mack T, Powell J and Whelan S (eds) *Cancer Incidence in Five Continents Vol V, 1987*. IARC Scientific Publications No. 88. IARC: Lyon

Narod SA, Stiller CA and Lenoir GM, (1991) An estimate of the heritable fraction of childhood cancer. *Br J Cancer* **63:** 993–999

Parkin DM and Stiller CA (1995) Childhood cancer in developing countries: environmental factors. *Int J Paed Haem Oncol* **2:** 411–417

Staines A (1997) The geographical epidemiology of childhood insulin dependent diabetes and childhood acute lymphoblastic leukaemia in Yorkshire. PhD thesis University of Leeds.

Staines AS, Bodansky HJ, McKinney PA, Alexander FE, McNally RJQ, Law GR, Lilley HEB, Stephenson C and Cartwright RA (1997) Small area variation in the incidence of childhood insulin-dependent diabetes mellitus in Yorkshire, UK: links with overcrowding and population density. *Int J Epidemiol* **26:** 1307–1313

StataCorp (1997) *Stata Statistical Software, Release 5.0*. Stata Corporation: College Station, TX

Stiller CA (1994) Population-based survival rates for childhood cancer in Britain, 1980–91. *Br Med J* **309:** 1612–1616

Stiller CA and Nectoux J (1994) International incidence of childhood brain and spinal tumours. *Int J Epidemiol* **23:** 458–464

Stiller CA, Allen MB and Eatock EM (1995) Childhood cancer in Britain: the national registry of childhood tumours and incidence rates 1978–1987. *Eur J Cancer* **31A:** 2028–2034

© Cancer Research Campaign 1998